# Editorial: Women in antimicrobial resistance and new antimicrobial drugs

**DOI:** 10.3389/fcimb.2023.1263568

**Published:** 2023-08-25

**Authors:** Rachel A. F. Wozniak, Rima El-Herte

**Affiliations:** ^1^ Department of Ophthalmology, University of Rochester School of Medicine and Dentistry, Rochester, NY, United States; ^2^ Department of Medicine, Division of Infectious Diseases, Creighton University School of Medicine, Omaha, NE, United States

**Keywords:** MDR, antimicrobial resistance (AMR) genes, antimicrobial resistance (AMR), isavuconazole, stewardship, novel antimicrobial

By 2050, it is projected that there will be 10 million deaths annually due to the rising incidence of antimicrobial resistance (AMR), outpacing diabetes and cancer (CDC, 2023). In addition to significant patient morbidity and mortality, AMR has a significant economic burden as the cost to treat infections caused by six multidrug-resistant germs can be more than $4.6 billion annually (Neloson et al., 2021).

As such, the World Health Organization (WHO) has already declared AMR as one of the top ten global health and development threats. Certainly, it is unanimously agreed that abuse, misuse, and overuse of antimicrobials are the driving factors to AMR. This is enhanced by suboptimal infection prevention/control, poor sanitation, and lack of access to clean water (WHO, 2015).

AMR is due to several factors identification of which largely now depends on molecular techniques.

Resistant pathogens are widely spread in the environment and not restricted to hospitals as demonstrated by Davidova-Gerzova et al. and Marchetti et al. Davidova-Gerzova et al. (“Hospital and community wastewater as a source of multidrug resistant ESBL- producing *Escherichia coli*”) examined a large collection of 408 *E. coli* strains collected from patients undergoing treatment for a urinary tract infection, raw hospital sewage, wastewater treatment plants inflows, outflows, and river samples upstream and downstream of wastewater treatment plants. Using whole genome sequencing, the authors performed comparative genomics analyses to compare the presence of antibiotic resistance genes, strain typing and presence of plasmids across these populations. Their studies highlighted the extremely high prevalence of antibiotic resistance across all isolates despite considerable other genetic variations.

Marchetti et al. (“Fosfomycin resistance mechanisms in Enterobacterales: an increasing threat”) explores the broad-spectrum antibiotic Fosfomycin regarding its mechanism of action and emerging antibiotic resistance. Fosfomycin is a broad-spectrum antibiotic that targets early stages of peptidoglycan formation and is currently FDA approved for urinary tract infections. With regards to antibiotic resistance mechanisms, bacteria either can develop a modification of the antibiotic target (MurA), reduced permeability or transport of Fosfomycin into the cell, or acquisition of antibiotic resistance genes. With regards to the acquisition of resistance genes, the FosA family of genes are metalloenzymes capable of disrupting the molecular structure of Fosfomycin, thereby inhibiting its activity. Currently there are eleven identified variants of FosA circulating globally, facilitated by its location on commonly found plasmids. Understanding the mechanisms of resistance is vital to improved surveillance and epidemiology studies as well as informing clinical care.

Efforts are also underway to discover new antimicrobials as these are natural products, derived from other microorganisms that produce these compounds to compete for limited environmental resources. While most ecological niches and commonly identified environmental microorganisms have been exploited, Abdelaziz et al. (“Bioactive metabolites of Streptomyces misakiensis display broad-spectrum antimicrobial activity against multidrug-resistant bacteria and fungi”) explores a subspecies of Streptomyces, S. misakiensis, to identify naturally occurring antimicrobial compounds that could be used to treat multidrug resistant bacteria and fungi. The authors purified 2 compounds, ursolic acid and tetradecamethylcycloheptasiloxne with broad spectrum antimicrobial activity in vitro. The authors go on to show that ursolic acid has antimicrobial activity in an in vitro septicemia model. While neither of these compounds are novel, as both have been identified and studied previously, the authors validate an approach to identifying novel natural products, and further supports the clinical development of ursolic acid.

To understand the epidemiology of resistant common mastitis strains, that has detrimental agricultural consequences, Shoaib et al. (Molecular epidemiology and characterization of antimicrobial-resistant Staphylococcus haemolyticus strains isolated from dairy cattle milk in Northwest, China) have found that the staphylococcus haemolyticus, the most common agent of cattle mastitis, have high resistance to erythromycin, trimethoprim-sulfamethoxazole and then ciprofloxacin; less to florfenicol, cefoxitin and gentamicin but not insignificant. Uniformly, they were susceptible to tetracycline, vancomycin, and linezolid. Multi drug resistant strains were also detected. The genes responsible for the antimicrobial resistance were various: ermA, ermB, ermC, ermF, erm(33), mphC, and msrB; vanA and vanB; cfr; cfxA; tetM, tetO, tetL, and tetK; fexA and floR; aacAaphD and aadD; gyrA, gyrB, grlA, and grlB; sul1, sul2, sul3, dfrA, dfrD, dfrG, and dfrK.

Despite the relentless pace at which bacteria develop resistance, unfortunately the development of novel therapeutics has been slow and extremely expensive. Since isavuconazole came to the market in 2015, antifungal agents Ibrexanfungerp and rezafungin were approved in 2021 and in 2023, respectively; the newest class of antimicrobial cefiderocol was approved in 2019. In his article, Zhang et al. (“Clinical research advances of isavuconazole in the treatment of invasive fungal diseases”) reports on the pharmacokinetics, drug-drug interactions, and clinical efficacy of the antifungal drug isavuconazole, a novel triazole antifungal agent used for the treatment of invasive aspergillosis, candidiasis and mucormycosis. It highlights that the isavuconazole has comparable efficacy to voriconazole with improved tolerability and safety. As such it has become a first-line therapy for the treatment of invasive aspergillosis and mucormycosis. Efficacy in cases of invasive candidiasis has also been demonstrated, however, isavuconazole is preferred as an alternative for de-escalation after first-line treatment. Altogether, this review demonstrates the favorability of isavuconazole as a novel therapy for invasive fungal diseases.

Investigators are now exploring artificial intelligence to aid in expanding the antimicrobial arsenal (Lepore et al., 2019; Melo et al., 2021) through using/modifying already existing molecules as well as develop new first-in-class drugs. Most recently bacteriophages were also used as a last resort to combat resistant organisms (Hatfull et al., 2022).

In addition to novel therapeutics, strict antimicrobial stewardship and monitored use of antimicrobials in humans, veterinary and agricultural fields are needed to combat antimicrobial resistance.

## Author contributions

RE-H: Conceptualization, Writing – original draft, Writing – review & editing. RW: Conceptualization, Writing – original draft, Writing – review & editing.
